# Trazodone augmentation could be a beneficial treatment for persistent insomnia in patients with comorbid PTSD and MDD that are good responders to escitalopram or sertraline: A one-group pretest-posttest study

**DOI:** 10.1192/j.eurpsy.2025.903

**Published:** 2025-08-26

**Authors:** V. Huljev, S. Nadalin, V. Kugelman, B. Aukst Margetić, Ž. Šurina Osmak, M. Vilibić

**Affiliations:** 1 Department of Psychiatry, Neuropsychiatric Hospital Dr Ivan Barbot, Popovača; 2 Department of Psychiatry, General Hospital “Dr. Josip Benčević”, Slavonski Brod; 3 School of Medicine, Catholic University of Croatia, Zagreb; 4 Department of Psychiatry, General Hospital Varaždin, Varaždin; 5 Department of Psychiatry, Dubrava University Hospital; 6 School of Medicine, University of Zagreb; 7 Ministry of Defence of the Republic of Croatia; 8 Department of Psychiatry, Sestre Milosrdnice University Hospital Centre, Zagreb, Croatia

## Abstract

**Introduction:**

Antidepressants are a valuable treatment option for patients with PTSD and MDD. Sleep is an important dimension of both: PTSD and MDD. Insomnia is a common residual symptom in patients with PTSD and MDD comorbidity who are generally good responders to SSRI monotherapy.

**Objectives:**

To explore wheather trazodone evening augmentation could be beneficial for persistent insomnia in patients with comorbid PTSD and MDD that are generally good responders to one of followed SSRIs: escitalopram or sertraline.

**Methods:**

Thirty outpatients (N=30) with comorbid war-related PTSD and MDD who are generally good responders to escitalopram (20 mg daily, N=15) or sertraline (100 mg daily, N=15) except persistent insomnia, were augmented with 100 mg trazodone. Trazodone was prescribed one-two hours before the sleep. Each patient met ICD-11 as well as DSM-V criteria for both: PTSD and MDD. No patient had psychiatric premorbidity, excluded by MINI. There was no significant somatic comorbidity that could influence the persistance of insomnia. Four sleep-related parameters were evaluated at two time points: at zero-point and 12-weeks after trazodone augmentation. This parameters/efficacy measures included: the improvement on the recurrent distressing dream item of the Clinician Administered PTSD Scale (CAPS-RDDI), reduction of the amount of time needed to fall asleep, prolongation of sleep duration, and reduction in the average number of arousals per night during the last seven days before assessment.

**Results:**

All sleep-related parameters improved significantly at the end of a 12-weeks follow-up: sleep duration prolongation (*p*<0.001), sleep latency decrease (*p*<0.001), median number of arousals per night decrease (*p*<0.001), and CAPS-RDDI median decrease (*p*<0.001).

**Conclusions:**

Trazodone augmentation could be a beneficial treatment strategy for persistent sleep problems in patients with comorbid PTSD and MDD who are generally good responders to escitalopram or sertraline monotherapy.

**Disclosure of Interest:**

None Declared

